# Peptide Deformylase (*def*) is essential in *Mycobacterium smegmatis*, but the essentiality is compensated by inactivation of methionine formylation

**DOI:** 10.1186/s12866-019-1611-7

**Published:** 2019-10-26

**Authors:** Noga Naor, Omer Gadot, Michal Meir, Daniel Barkan

**Affiliations:** 1 Koret School of Veterinary Medicine, The Robert H. Smith Faculty of Agriculture, Food and Environment, The Hebrew University of Jerusalem Rehovot Campus, Rehovot, Israel; 2 The Ruth Rappaport Children’s Hospital, Rambam Health Care Campus, Haifa, Israel

**Keywords:** Mycobacteria, Protein synthesis, Co-translation, Peptide Deformylase, Formyl Transferase

## Abstract

**Background:**

Co-translational processes in bacteria are attractive drug targets, but while some processes are essential, others are not. The essentiality of Peptide Deformylase (PDF, *def*) for vitality of mycobacteria was speculated, but never unequivocally proven.

**Results:**

Here we show by targeted deletion experiments that *def* can only be deleted from *M. smegmatis* when an additional copy is present; that prior deletion of tRNA^fMet^-Formyl Transferase (FMT, encoded by *fmt*) renders *def* completely dispensable; and that re-introduction of *fmt* into a *Δdef* mutant is not possible – constituting a definitive proof for the essentiality of *def* in mycobacteria.

**Conclusions:**

Peptide deformylase is essential in *M. smegmatis*, but the fact that inactivation of *fmt* renders the gene completely dispensable, and thus any inhibitor of *def* useless, casts doubt on the usefulness of PDF as a drug-target in mycobacteria.

## Background

Mycobacteria continue to be a major health threat throughout the world. *M. tuberculosis* causes over 1.6 million deaths worldwide [1], but the burden of *M. leprae* and *M. ulcerans* (mostly in the developing world), and that of pathogenic rapidly-growing mycobacteria such as *M. abscessus* and *M. kansasii* is rising as well [2–4], mostly in the developed world. Many mycobacteria exhibit substantial drug resistance and tolerance, making the need for novel therapeutics more urgent [5]. Understanding of physiologic processes unique to bacteria is important for identifying essential pathways that could be targeted. One such process is the prokaryote-specific formylation and de-formylation of the methionine that starts the synthesis of every protein [6, 7]. In mycobacteria, methionine is first formylated by tRNA^fMet^-Formyl Transferase (FMT, encoded by *fmt*), and only a formylated methionine can be used as the first amino acid in a new protein [8, 9]. Shortly after it emerges from the ribosome, the leading methionine is de-formylated by Peptide Deformylase (PDF, encoded by *def*). Following deformylation, methionine is removed from the majority of mycobacterial proteins by either Methionine Amino-Peptidase A (MetAPa, encoded by *mapA*) or MetAPc (encoded by *mapB*) [8, 10]. FMT was suggested to be essential by a transposone-mutant library analysis in *M. tuberculosis* [11–13], but directed deletion experiments showed it to be completely dispensable for *M. smegmatis*, and to cause only mild growth retardation in *M. bovis* when deleted [9]. In MetAP’s, chemical inhibition studies first suggested *mapA* was essential whereas *mapB* was not [8], but directed gene deletion experiments on *M. bovis* proved it to be the other way around – *mapB*, and not *mapA*, was the essential one, with some redundancy [10].

PDF (and *def*) is postulated to be vital in all bacteria – probably as MetAP’s cannot function on proteins where the formyl group was not removed [14]. *def* was suggested to be essential in *M. bovis-BCG*, and a chemical inhibitor of it had antibacterial activity [15]. However, given the fact chemical inhibition experiments, as well as the genetic method used to prove essentiality in that study, may mistake a slow-growth phenotype for a lethal one, thus leading to incorrect essentiality attributions (as was the case for *fmt* and *mapA/mapB*). We opted to definitively examine PDF’s role in mycobacterial viability by deletion experiments, as well as deletion on an *fmt*-null background.

## Results

### *def* cannot be deleted from the chromosome of *M. smegmatis*

**Fig. 1:**
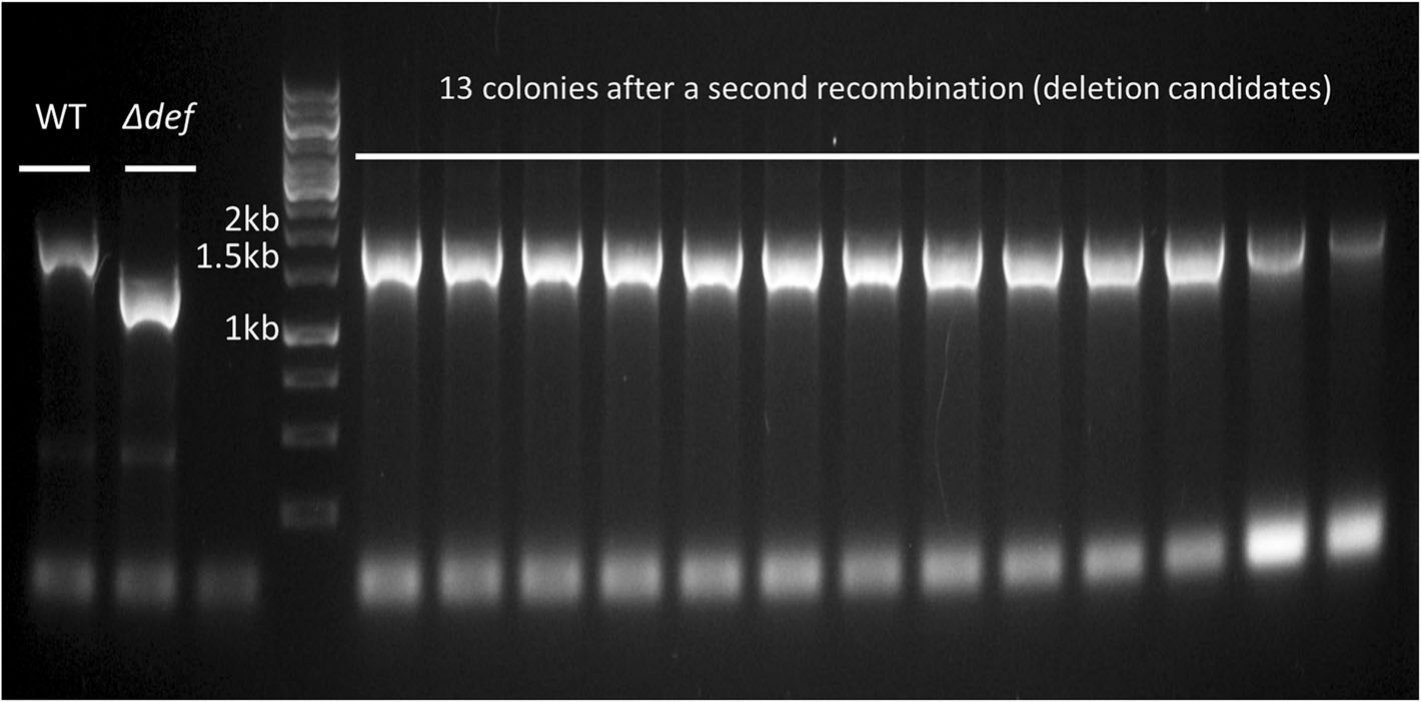
*def* cannot be deleted from wt *M. smegmatis*. On the right, colonies after a confirmed second recombination event were checked by PCR to differentiate *def*-deletion (resulting in 1.2 kb product) from reversal to wt (1.8 kb fragment). 13 colonies are shown, out of 60 examined. On the left – wt control, and a true *Δdef* mutant (obtained as described in section 3.2)

For the deletion of *def*, we used the two step allelic exchange system, for which we constructed the plasmid pDB354. The plasmid contains both *zeocin*^*R*^ and *streptomycin*^*R*^ resistance genes (for positive selection), *sacB* (for negative selection), and the two flanking regions of *def* (~ 600 bp each, placed adjacent to each other). The plasmid was electroporated into wt *M. smegmatis*, colonies were selected on zeocin and streptomycin, and examined for successful recombination at one of the *def* flanking regions by PCR. One correct colony was found, and named *M.smeg*^*int*^ (for “intermediate”). These bacteria were grown without antibiotic selection for 15 generations to allow for a second recombination event, and plated on sucrose 10% for negative selection. One hundred colonies that grew on sucrose were patched on either no selection or zeocin/streptomycin to differentiate true second recombination events from *sacB* inactivating mutations. Sixty colonies were found to be true second recombination events (sucrose^R^/Zeocin^S^/streptomycin^S^). All were examined by PCR that produces an 1800 bp fragment in the case of reversal to wt, and 1200 bp in case of a *def* deletion genotype. All 60 colonies were found to revert to wt, with none having the deletion genotype (Fig. 1). We therefore conclude that deletion of *def* is, at the very least, highly deleterious in wt *M. smegmatis*.

### In the presence of an additional copy, *def* can be deleted

**Fig. 2:**
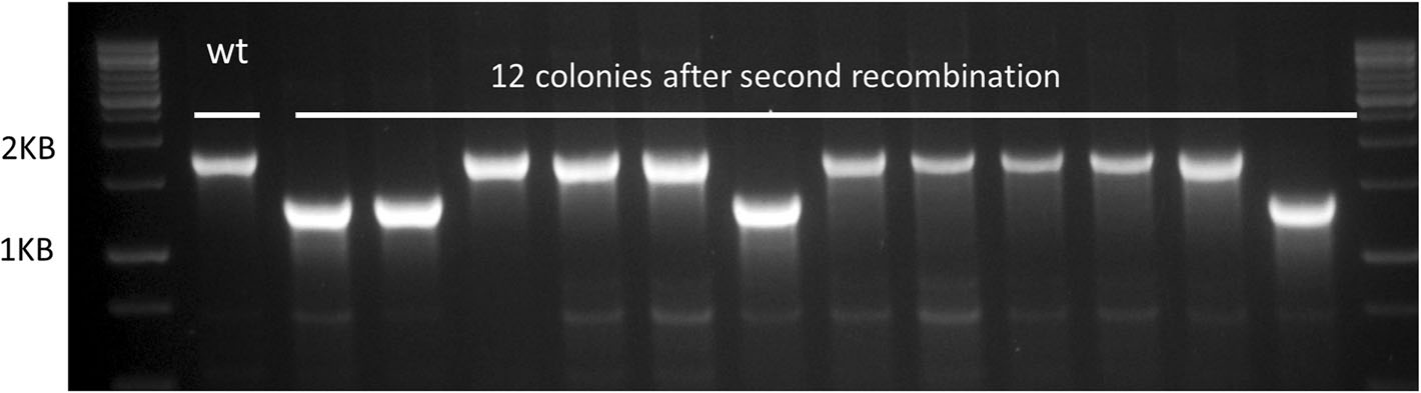
With pre-complementation with an additional copy of *def*, a deletion of the native gene is easily obtainable. Right – 12 colonies after second recombination. 4 of them have the PCR product obtained from a *Δdef* genotype (1200 Bp). Left – wt control (1800 bp)

We complemented *M.smeg*^*int*^ with an additional copy of *def* at the *attB* site, on a hygromycin-selected plasmid. We then proceeded to the second recombination as described in the previous section. This time, out of the 12 colonies that underwent a second recombination event, four were *def* deletions, whereas the other eight reverted to wt genotype (Fig. 2), indicating the in the presence of an additional copy of *def*, the native *def* is completely dispensable (and that the deletion strategy used was valid).

### The lethality of *def* deletion is compensated by tRNA^fMet^-Formyl Transferase (FMT, *fmt*) inactivation

**Fig. 3:**
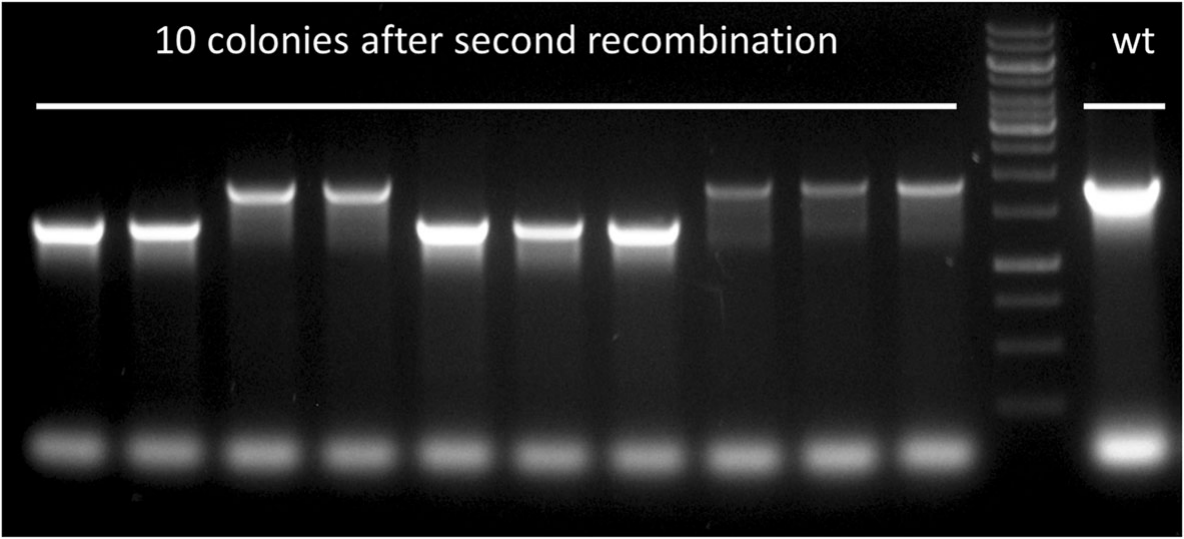
On a *Δfmt* background, deletion of *def* is highly efficient. Left: 10 candidate colonies. Right: wt control. The leftmost colony was later re-named mDB224

The physiologic function of the *def* product (Peptide Deformylase, PDF) is to remove the formyl group from the leading methionine. Since this formyl group is originally attached by another enzyme, the tRNA^fMet^-Formyl Transferase (FMT, encoded by *fmt*), we speculated that on an *fmt*-deletion background, *def* may become dispensable. We previously described a complete deletion of *fmt* in *M. smegmatis* (strain mDB22) and in *M. bovis* [9]. Contrary to previous reports, the deletion of *fmt* was not lethal, causing only a trivial growth defect in *M. smegmatis*, and a 2–3 fold slower growth in *M. bovis* [9]. We decided to examine if *fmt* deletion renders *def* completely dispensable. We created an mDB22^int^ strain using pDB354 (as was done for *def* deletion on wt background), proceeded to second recombination, and then examined colonies using the previously described PCR. This time we easily obtained a deletion mutant, with 5 colonies out of 10 candidates being *def* knock-outs (Fig. 3). This confirms that in the absence of *fmt*, *def* is completely dispensable. One of these colonies was arbitrarily chosen for continued work, and named mDB224 (*M. smegmatis Δdef/Δfmt*).

### *fmt* is incompatible with a *Δdef/Δfmt* genotype

**Fig. 4:**
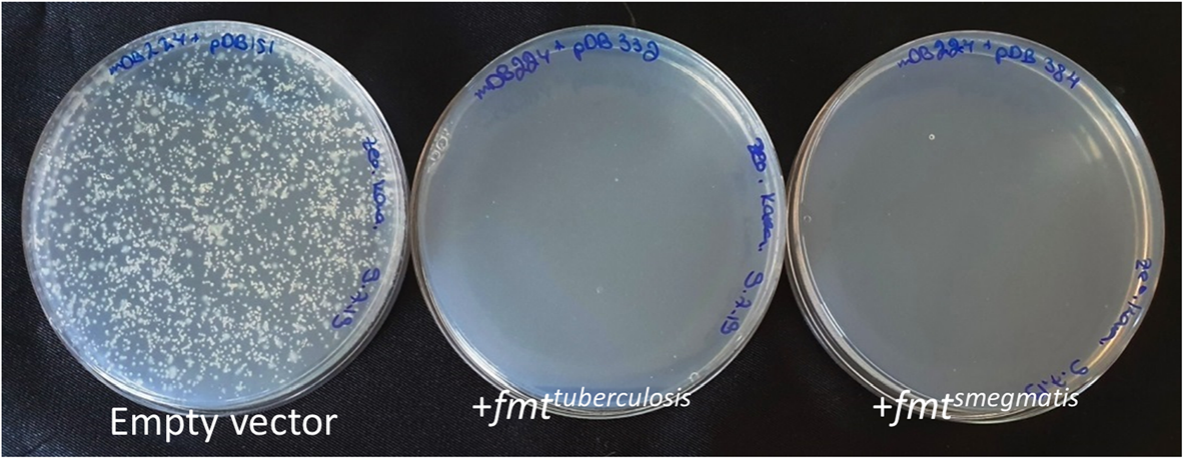
A functional *fmt* is incompatible with a *Δfmt/Δdef* genotype. mDB224 (*Δfmt/Δdef)* was electroporated with a control vector (left), control+*fmt*^*tuberculosis*^ (middle), and control+*fmt*^*smegmatis*^ (right). The picture was taken after 4 days incubation, but plates were kept for 10 days before discarding, not to miss slow-growing mutant

The fact *def* could only be deleted from *M. smegmatis* (*and M. bovis*, previously) when an additional copy was present at the *attB* site, does not constitute a proof it is completely essential – as a viable, but with a substantial growth defect mutant, would not be found in these types of experiments. We therefore wanted to examine if *fmt* could be re-introduced into a double *def/fmt* null mutant. If *def* is completely essential (on an *fmt*-positive background), then this re-introduction would yield no colonies. Slow-growing mutants will be discovered, as there are no normal-growing bacteria to mask them. We therefore prepared two genetic constructs to re-introduce *fmt* into the *fmt/def* double deletion mutant (mDB224): *fmt*^*smegmatis*^ (pDB384) or *fmt*^*tuberculosis*^ (pDB332) on a multi-copy episomal vector. Both plasmids had a stringent double selection (zeocin and kanamycin) to prevent background colonies. All electroporations were done with an empty vector control (also expressing mCherry, with resulting pink colonies). Whereas the control yielded multiple colonies, the *fmt*-containing plasmids yielded none (Fig. 4). Insertion of both pDB332 and pDB384 into wt *M. smegmatis* yielded multiple colonies (data not presented), showing the plasmids themselves are transformable, and confer antibiotic resistance as expected. We therefore conclude that *fmt* re-introduction is incompatible with a *def*-deletion background, providing unequivocal proof of *def* essentiality for viability, and not just for normal or near-normal growth.

## Discussion

Protein synthesis and co-translational processes are an attractive target for development of antibacterials, as there are several steps quite unique to prokaryotes – specifically the formylation and de-formylation of the leading methionine [6, 7]. However, targeted enzymes should be those shown to be essential for viability, and the essentiality is sometimes difficult to prove. *Fmt* was previously claimed to be essential, based on transposon-mutant library analyses [11–13]. However, these analyses are strongly biased against viable but slow-growing mutants, and directed deletion experiments in *M. smegmatis* and in *M. bovis* unequivocally showed the gene was dispensable [9]. Similarly, a chemical inhibition assay suggested MetAP1a was essential in *M. tuberculosis*, and that MetAP1c was not [8]. Again, directed deletion experiments showed the opposite [10]. As opposed to the well-established, complete essentiality of MetAp’s in all life forms [17, 18], the role of DEF in different organisms is deemed controversial [19]. A targeted deletion in *M. bovis-BCG* only yielded colonies when an additional copy of the gene was present (in a two-step allelic exchange technique), leading to the conclusion *def* was essential (as also expected from what is known in other bacteria) [15]. However, these analyses are based on testing of several dozen colonies appearing on a negative-selection plate. Even if the gene in question is completely dispensable, at least 50% of colonies will revert to wt, and if the deletion of the gene is viable but causes growth retardation, the proportion of the deletion mutants will become much smaller, as the wt colonies will appear well before the deletion ones. Therefore, this kind of experiment can suggest an important role in growth, but not absolute essentiality. Here, we first repeated the previous path, where we showed the gene could be deleted when an additional copy was present, and no deletion mutants could be obtained when no pre-complementation was done. We then showed that on a background of *fmt* deletion, the role of *def* becomes completely insignificant, as evident by a 50% deletion rate versus reversal to wt. However, we then showed that re-introduction of *fmt* (both *smegmatis* and *tuberculosis* origin) is impossible into a mutant that has no *def* – meaning a functional *fmt* is incompatible with a *Δdef* genotype – and this constitutes a definitive proof of *def* essentiality.

Why deformylation is essential is not completely clear. One can postulate the formyl group blocks the activity of the methionine aminopeptidase enzymes (MetAP), who’s activity is known to be essential in all life forms [17], including prokaryotes. One should remember the leading methionine is not removed from all synthesized proteins, and it is possible that in proteins retaining this methionine, the formyl group may be retained as well, with no compromise of viability. This may explain how mitochondrial proteins in eukaryotes constantly retain the formyl group [18, 20, 21]. Nonetheless, genes homologous to *def* were shown to exist in the nuclear genomes of higher eukaryotes, together with studies demonstrating mitochondrial DEF expression with highly selective functions in human cells [19, 22, 23]. This suggests DEF does play a role in specific deformylations in eukaryotic organisms as well. Moreover, it was shown that PDF of human origin could compensate for *def* deficiency in an *E. coli* mutant with conditional expression of *def* [24], pointing the conjecture that functionally, human PDF might be similar to the bacterial one.

The essentiality of *def* raises the question of whether it can serve as an effective drug target. A previous study showed chemical inhibition of *def* does have antimycobacterial properties [25]. However, it appears from our study that in the case of *fmt* inactivation, the inhibition of *def* becomes irrelevant to growth. The barrier for spontaneous resistance mutations is therefore relatively low, as inactivating mutations (unlike mutations that retain activity, but do not bind an inhibitor) are fairly common. It remains to be seen if the co-translation pathway is indeed a viable drug target.

## Conclusions

PDF (*def*) was unequivocally shown to be essential in *M. smegmatis*, and probably in all mycobacteria. However, as genetic inactivation of FMT (*fmt*) rendered the bacteria completely neutral as for the existence or absence of PDF, the role of PDF as a viable drug target remains questionable. It could, however, be part of a combination regimen, with other drugs that act on non-related targets.

## Methods

### Strains and growth conditions

*Mycobacterium smegmatis* (*mc*^*2*^*–155*) were grown in Middlebrook 7H9 media, supplemented with 0.5% glycerol, 0.5% dextrose, and 0.02% Tween80. For solid agar plates, we used 7H10 Middlebrook with glycerol and dextrose, but no tween. Antibiotic concentrations were: kanamycin 40 μg/mL, zeocin 25 μg/mL, streptomycin 20 μg/mL, and hygromycin 50 μg/mL (150 μg/mL for *E. coli*). *Δfmt M. smegmatis* was previously described [9].

### Deletion and complementation of *def*

Deletion was done in the two-step allelic exchange, as previously widely described [16]. The plasmid used for the first step recombination was pDB354. The upstream and downstream flanking regions of *def* were PCR amplified from *M. smegmatis* genome, fused into a single PCR product using primers defsmko1–5, and cloned into a plasmid containing *zeocin*^*R*^, *streptomycin*^*R*^ and *sacB*. After the second recombination, to differentiate between a deletion mutant and reversal to wt, we used primers defKOchqD and defKOchqU that produce an 1800 bp fragment in wt, and 1200 bp fragment in a deletion mutant. To verify the 1200 bp product is indeed the result of a successful deletion, the PCR product was sent for Sanger sequencing, and was shown to be identical to the wt-genotype, 1800 bp product at both ends, but to lack the bulk of the def gene, as constructed in the plasmid used for the deletion (pDB354), resulting in shortening by ~ 600 bp. To complement with an additional copy of *def*^*smegmatis*^, *def* was amplified using primers defSmU and defSmD, and cloned into the integrating plasmid pYUB412. All the primers sequences are shown in Additional file 1: Table S1.

### Complementation with *fmt*

A multicopy episomal plasmid, selected by zeocin and kanamycin, and also expressing mCherry, with a copy of *fmt*^*tuberculosis*^ was previously described by us (pDB332, 9]. A similar plasmid (with no mCherry) for *fmt*^*smegmatis*^ was constructed using primers fmt smg_R_xbaI, and fmt smg_BamHI, and cloned into pDB151, producing pDB384. Electroporations were done as previously widely described.

## Supplementary information

**Additional file 1: Table S1.** Primers (oligonucleotides) used in this study.

## Data Availability

No large databases were used for this study. The datasets used and/or analyzed during the current study are available from the corresponding author on reasonable request.

## Abbreviations

FMT: Formyl methionine transferase
MetAP: Methionine aminopeptidase
PDF: Peptide deformylase
WT: Wild type
