# Supplementary material for: Peptide Deformylase (def) is essential in Mycobacterium smegmatis, but the essentiality is compensated by inactivation of methionine formylation
Source: BMC Microbiol. 2019 Oct 26;19:232. doi: 10.1186/s12866-019-1611-7 (PMC6815462; doi:10.1186/s12866-019-1611-7)
Supplement: Supplementary file 1 — Additional file 1: Table S1. Primers (oligonucleotides) used in this study. [file 12866_2019_1611_MOESM1_ESM.pdf]

Supplementary Table 1:

Primers (oligonucleotides) used in this study:

Oligo nucleotide name	Sequence 5'=>3'
defsmko1	AGTCTAGAATCCGAGGAGTCGTCGTCGGAACCCA
Defsmko2	TGTGCAGTACTGGGTCTCCGACAATG
Defsmko3	CAAGAAGGCCGTCAAACGCAACGGC
Defsmko4	ATGCGGCCGCCACGAACTGCGCATCGATGTTCCGGC
Defsmko5	GCATTGTCGGAGACCCAGTACTGCACACAAGAAGGCCGTCAAACG
defKOchqD	GACCTCGACCGGCACCTTGCGTTCG
defKOchqU	CTCGGACGTCTTGGTGGGTGTGGTC
defSmU	TCAGATCAGAGTCGTCACCGGATTG
defSmD	AGCTCAGACATCAGGCACCTCGGC
fmt smg_R_xbaI	TCTAGAGCGGACGTTTGCGCGGCGGG
fmt smg_BamHI	GGATCCGCTGTCGCTCGGCTCGTCCT
